# Transcriptional profiling of mesenchymal stromal cells from young and old rats in response to Dexamethasone

**DOI:** 10.1186/1471-2164-7-95

**Published:** 2006-04-27

**Authors:** Uri David Akavia, Irena Shur, Gideon Rechavi, Dafna Benayahu

**Affiliations:** 1 Department of Cell and Developmental Biology, Sackler School of Medicine, Tel Aviv University, Tel Aviv 69978, Israel

## Abstract

**Background:**

Marrow-derived stromal cells (MSCs) maintain the capability of self-renewal and differentiation into multiple lineages in adult life. Age-related changes are recognized by a decline in the stemness potential that result in reduced regeneration potential of the skeleton. To explore the molecular events that underline skeletal physiology during aging we catalogued the profile of gene expression in *ex vivo *cultured MSCs derived from 3 and 15 month old rats. The *ex vivo *cultured cells were analyzed following challenge with or without Dexamethasone (Dex). RNA retrieved from these cells was analyzed using Affymetrix Gene Chips to compare the effect of Dex on gene expression in both age groups.

**Results:**

The molecular mechanisms that underline skeletal senescence were studied by gene expression analysis of RNA harvested from MSCs. The analysis resulted in complex profiles of gene expression of various differentiation pathways. We revealed changes of lineage-specific gene expression; in general the pattern of expression included repression of proliferation and induction of differentiation. The functional analysis of genes clustered were related to major pathways; an increase in bone remodeling, osteogenesis and muscle formation, coupled with a decrease in adipogenesis. We demonstrated a Dex-related decrease in immune response and in genes that regulate bone resorption and an increase in osteoblastic differentiation. Myogenic-related genes and genes that regulate cell cycle were induced by Dex. While Dex repressed genes related to adipogenesis and catabolism, this decrease was complementary to an increase in expression of genes related to osteogenesis.

**Conclusion:**

This study summarizes the genes expressed in the *ex vivo *cultured mesenchymal cells and their response to Dex. Functional clustering highlights the complexity of gene expression in MSCs and will advance the understanding of major pathways that trigger the natural changes underlining physiological aging. The high throughput analysis shed light on the anabolic effect of Dex and the relationship between osteogenesis, myogenesis and adipogenesis in the bone marrow cells.

## Background

The stromal compartment of the bone marrow contains mesenchymal stem and progenitor cells with high proliferating capacity in addition to cells at different stages of maturation. The mesenchymal cells are at the front of cell research today which differentiate *in vitro *and *in vivo *to multiple lineages including fibroblasts, adipocytes, cartilage, myogenic, and osteogenic cells [1-7]. MSCs also harbor the potential of trans-differentiation to many different lineages, thus providing a possible source of progenitors for cell therapy and tissue repair including bone, cartilage, cardiac, pancreas regeneration and neural injury repair [8]. Cell differentiation through distinct maturational stages involves coordination and activation of different sets of genes. Progenitor cells derived from the stroma compartment of the bone marrow differentiate under the control of transcription factors, which serve as lineage specific master genes for discrete differentiation steps. Definition of the key differentiation signals is important in order to induce the desired *ex vivo *lineage-specific maturation pathways.

Age related hormonal changes, for example a decline in sex hormones levels, are associated with a decrease in the number and activity of osteogenic cells and an increase in numbers of adipocytes [9-15]. It is generally accepted that these changes arise from a decrease in the stemness potential accompanied by a decrease in the proliferative ability and osteogenic capacity of the bone marrow cells [11,15-18]. The changes in stemness with age result in reduced osteogenesis and increased adipogenesis, affecting the skeletal structure and the immune system. Age-related changes associated with osteoporosis were previously studied by us in animal models [15,19] and in *ex vivo *cultures of stromal cells [20]. It is clear that the physiological status of the body affects the skeleton at the cellular level, but the underlying molecular mechanism remains unresolved.

The action of native or pharmacological glucocorticoid hormones, such as Dexamethasone (Dex), is mediated via glucocorticoid receptors (GRs). Dex is recognized by multiple effects on a wide range of tissues and physiological conditions in the body [21]. Dexamethasone promotes osteogenesis *in vitro *[22], and induces the expression of osteogenic markers in MSCs [20,23-25].

In this study, we analyze the molecular changes in aged rats that influence the cellular potential and the response to Dex. We used GeneChip technology to explore the molecular changes regulating the processes that govern the commitment and differentiation of the MSCs in young and aged animals. This approach enables us to analyze genome-wide patterns of mRNA expression and to provide an efficient access to genetic information. We compared gene profiling between MSCs cultured *ex vivo *from young and old rats (3 and 15 month old). The cells were maintained *in vitro *and maintained in presence or absence of Dex. The RNA extracted was analyzed to assess the transcriptome profile of MSCs. From the microarrays we further analyzed the lineage-specific gene expression in the MSCs enabling us to reveal genome-wide patterns of mRNA expression and to sort the gene profiles that govern various cell activities.

## Results

### The GeneChip analysis

Primary marrow stromal cells (MSCs) derived from the bone marrow include stem and progenitor cells with high proliferating capacity. We have earlier studied MSC in rat [19] and mouse [4,15] models and demonstrated that the decline in stemness with aging is associated with bone atrophy, increase of adipocytes and augmentation of T-lymphopoiesis.

The present study aimed to compare the profile of genes expressed by cultured MSCs derived from 3 and 15 month old rats, expanded *in vitro *and treated or untreated with Dex. We used microarray technology to compare the transcriptome profile between cultured MSCs from 4 experimental groups: young Y (3 month) and old O (15 month) rats that were treated (YT, OT) or untreated (YU, OU) with Dex. The molecular analysis represents snapshot of cellular states from these rats and reflects their potential for tissue differentiation. The analysis resulted with 10290 Probe Sets (PS) that changed with fold change of two or higher. The PS clustering attempts to elucidate the pattern of gene expression which is unique to MSCs.

### Genes differentially expressed between experimental groups

The gene expression was studied for RNA from each age group, and the overlap between them was calculated. RNA was retrieved from four groups of cells according to treatment and animal age: untreated cells from 3 month old rats (young untreated – YU – 1), 15 month old rats (old untreated – OU – 2) and treated cells from young and old rats (YT – 3, OT – 4). Differentially expressed PS were marked either as increasing or decreasing according to expression ratios between Dex-treated and untreated cells. In cells derived from young rats, the analysis resulted in 3318 differentially expressed PS, 1604 increasing (YI) and 1714 decreasing (YD). In cells derived from old rats, 2725 PS were differentially expressed – 1314 increasing (OI), 1411 decreasing (OD) following treatment.

**Figure 1:**
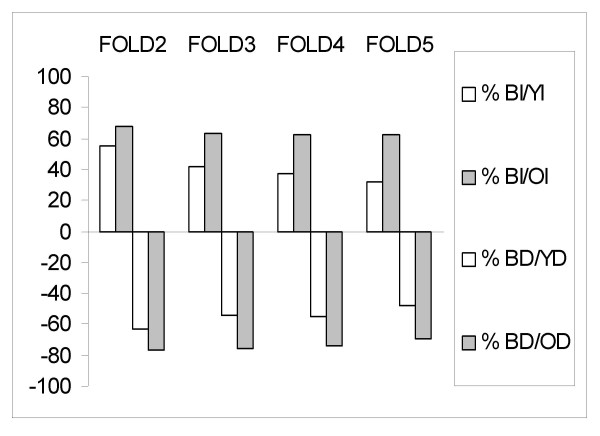
**Ratio of PS differentially expressed in both age groups vs. each age group**. BI – PS that increased in cells derived in both age groups, YI – PS that increased in cells derived from young rats, OI – PS that increased in cells from old rats. BD, YD, OD – PS that decreased in cells from both age groups, in cells from young and old rats respectively. The graph presents ratios for multiple fold changes: 2,3,4,5. BI/YI and BD/YD (white bar), BI/OI and BD/OD (grey bar).

A total of 888 PS increased and 1077 PS decreased in cells derived from both young and old rats due to the Dex treatment. The analysis of differentially expressed PS was repeated for fold change of 3, 4 and 5 and resulted in smaller numbers of differentially expressed PS. The number of PS differentially expressed at fold change higher then 2 decreased, but the ratio of expressed PS in both ages versus PS expressed in cells derived from young or old rats did not change much, indicating a common response to Dex in both ages (Figure 1).

The ratio of PS changed in both groups relative to PS changed in cells from old rats remained constant. The ratio of PS increased in both groups (BI) relative to PS increased in old (OI) and the ratio of decreasing PS (BD/OD) remained at about 60% and 70%, respectively. The PS increased in both groups (BI) relative to PS increased in young (YI) lowered from 55% to 32% as the number of differentially expressed PS decreased (due to increasing fold change). The ratio of PS decreased in both groups (BD) relative to PS that decreased in young (YD) lowered from 63% to 48% as the number of PS in general decreased.

### Clustering analysis

**Figure 2:**
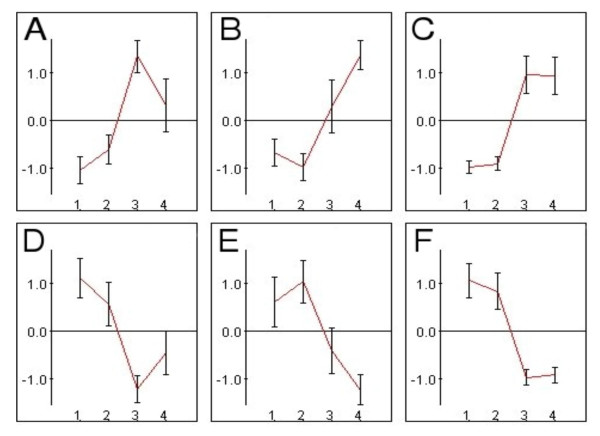
**Illustration of the clusters A to E**. Four groups of analyzed samples are presented: 1 – young untreated (YU), 2 – old untreated (OU), 3 – young treated (YT), 4 – old treated (OT). The mean of each PS was standardized to 0, and the standard deviation was 1. The clusters included PS increased in cells from young rats (A), increased in cells from old rats(B), increased in cells from both age groups (C), decreased in cells from young rats(D), decreased in cells from old rats (E), decreased in cells from both age groups (F). Expression patterns of all clusters are presented with error bars. PS at levels lower than zero indicates the repressed expression and PS at levels above zero indicates the induced expression.

PS were clustered to profile distinct Dex effect for both age groups; 4060 PS were differentially expressed, up or down regulated more than 2-fold, due to the Dex treatment in cells derived from young and old rats. The analysis resulted in six clusters that were divided into PS that increased (A-C) or decreased (D-F) (Figure 2). Cluster A (704 PS) represents PS that increased in cells derived from young rats, Cluster B, represents 420 PS increased in old rats, and PS that increased in cells from both age groups are included in Cluster C (888 PS). PS that decreased in cells derived from young rats are put in Cluster D (631 PS), PS that decreased in cells from old rats – in Cluster E (322 PS), and Cluster F (1071 PS) presents PS that decreased in cells from both age groups. A small number of PS increased in cells from young rats and decreased in cells from old rats (12 PS) or the opposite pattern of expression (6 PS) (data not shown). PS who had a known Gene ID were analyzed for functional properties of the genes.

### Functional pathways involved in mesenchymal cells differentiation

Comparing gene expression profiles between Dex-treated or untreated cultured cells obtained from young and old rats results with clustering of 6 groups (Figure 2) analyzed for statistically significant functions, using the GOTM. This analysis shows functions affecting response to Dex and differentiation of skeletal and bone marrow cells. Tables 1 and 2 describe the major functions that have a high fold change and highlight various differentiation pathways of mesenchymal cells affected by Dex treatment.

Series of genes increased following Dex treatment of MSCs are represented in clusters A-C (Figure 2): genes increased in cells derived from young rats (A), in cells derived from old rats (B) and genes increased in cells derived from both age groups (C) (Table 1). The overall gene expression reflects the Dex-related shift of cellular metabolism, which results in a decrease in proliferation, coupled with differentiation of MSCs to osteogenic and myogenic directions and a repression of the adipogenic pathway.

Cluster A (genes that increased only in cells derived from young rats) includes *VEGF*, *Id1*, *Madh5 *and *beta-catenin *which are related to angiogenesis (p = 0.00125), implying that neovascularization is linked to osteoblast maturation and bone remodeling (p = 0.00448), which included the genes *PTHr1*, *IBSP*, *Madh5 *and bone ECM protein *Mepe*. These genes are related to osteogenesis and indicate an osteogenic differentiation of these cells. Human and mouse orthologs disclosed components of ECM structural constituent such as *collagens IV, V, VI *and *laminin *(p = 0.00287). Dex has an anabolic effect on cells from young rats that increases osteogenesis-related markers, while such an effect was undetected in cells derived from old rats and is possibly related to the decrease in bone formation associated with aging.

Cluster C presents genes that increased due to Dex treatment in cells derived from both young and old rats (Table 1) which are related to cell growth (p = 2.36E-06), regulation of cell cycle (p = 0.00418), muscle differentiation and contraction (p = 0.00555, p = 0.00157), cytoskeletal components (p = 0.00868) including structural constituents of the cytoskeleton and actins (p = 0.00533, p = 0.00803). A reciprocal relationship of proliferation and differentiation is recognized by a decrease in genes that activate proliferation as cells differentiate. Human and mouse orthologs of the rat genes are related to the dystrophin complex (p = 0.00311) which plays a role in development of skeletal muscle. An increase in muscle related genes due to Dex indicates the potential of mesenchymal stem cells to differentiate into myotubes.

Genes decreased following Dex treatment are clustered in D-F (Figure 2) for cells derived from young rats (D), old rats (E) and from both age groups (F) (Table 2). Genes repressed following Dex treatment in cells derived from young rats are related to adipocyte differentiation, regulation and function (Table 2, Cluster D). *PPARγ *and *CEBPα *are TFs essential for adipocyte differentiation (p = 0.0056). *PPARγ *maintains adipogenesis affecting early differentiation and survival of mature adipocytes and *CEBPα *is activated later than *PPARγ*. An inhibition of *PPARγ *and *CEBPα *in response to Dex results with a decrease of adipogenesis. The effect of Dex in lowering the adipogenesis (Table 2, cluster D) is complementary to increasing the osteogenesis (Table 1, cluster A).

Cluster F (Table 2) summarizes genes repressed by Dex treatment in cells derived from both age groups and includes genes related to defense response (p = 0.0006). We noticed a decrease in RAS signaling genes (p = 0.00016), such as *Nras*, *Kras2*, *Grb2 *and *Aps*, which is complimentary to the proliferation and differentiation genes induced by Dex (Table 1, Cluster C). Dex repressed genes related to catabolism (p = 0.00027) and proteolysis (p = 0.00312), including mainly lysosomal (p = 1.4E-07) hydrolases (p = 0.00019). The suppression of adipogenic differentiation is monitored by the decrease of genes related to lipid binding (p = 0.00024), lipid transport (p = 0.0096) and diacylglycerol binding (p = 0.00474) including genes such as *FABP-4 *(*aP-2*) and *lipoprotein lipase *(*Lpl*). Protein Kinase C isoforms comprised some of the genes identified as lipid binding cluster. The variants of this gene have a role in muscle differentiation and mediate PTH receptor action in repressing adipogenesis and inducing osteogenesis.

**Figure 3:**
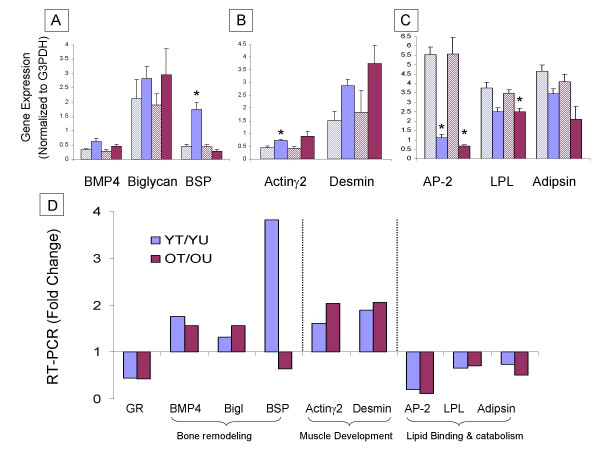
**RT-PCR analysis of genes related to bone remodeling, muscle development and lipid binding and metabolism**. (A) – bone remodeling include BMP4, Biglycan, BSP, (B) – muscle development include Actin, Desmin and (C) – lipid binding and metabolism include AP-2, LPL, Adipsin. (A-C) – Y axis represents the gene expression level (normalized to G3PDH) of the YU (light blue bars), YT (blue bars), OU (pink bars), and OT (purple bars). T-test comparing expression level in treated and untreated age-matched samples was performed – * p value < 0.005, expression levels of all genes analyzed except Biglycan are significant with p value < 0.05. (D) – Y axis represents the ratio between treated and untreated cells from young (blue bars) and old (purple bars) rats.

The functional analysis of the clusters resulted in major pathways – an increase in osteogenesis and muscle formation, coupled with a decrease in adipogenesis. We selected the genes representing these differentiation pathways (Table 3) and divided them into clusters and functional groups. Genes which increased only in cells from young rats belong to the functional group of bone remodeling and angiogenesis. Genes that increased in both cells derived from young and old rats are related to muscle development and cell cycle. Other genes that decreased only in cells derived from young rats are related to adipocyte differentiation and genes that decreased in both groups are related to lipid binding, catabolic peptidases, immune response and RAS signaling. To validate the results from the gene array we employed RT-PCR analysis summarized in Figure 3 (p values are specified in the legend). Analyses were performed for the expression of *BSP*, *Biglycan *and *BMP4 *to follow osteogenic pathway; *desmin *and *actin γ2 *– to follow the effect of age and Dex on the cell potential to differentiate to myoblasts. *AP-2*, *LPL *and *Adipsin *were used to follow adipogenesis. Analyzed markers showed age and Dex related changes detected with a similar fold change levels for both RT-PCR and array analysis. The expression of *GR *was analyzed to verify that the response to Dex resulted with down regulation of the receptor. The expression level of *GR *decreased in cells from both age groups – the ratios were 0.4 and 0.54 on the chip, and 0.44 and 0.42 by RT-PCR. The fold change for desmin on the chip array was 2.4 for cells derived from young rats and 2.36 for cells derived from old rats and 1.9 and 2.05 by RT-PCR, respectively. *AP-2 *expression decreased on the chip (0.04 and 0.01), and by RT-PCR (0.2 and 0.12). Dex triggered an increase in the osteogenic related genes that was more prominent in young animals. An increase was noted in muscle-related genes in cells from both age groups; and a decrease in genes related to the lipid metabolism. The expression level of these genes behaved in a similar manner on the chip and when checked by RT-PCR. The comparison between the two methods confirmed the biological effect of Dex on cells from young and old rats.

## Discussion

Mesenchymal stem cells have an ability to differentiate into multiple lineages including adipocytes, myocytes and bone forming cells [1-7]. Aging affects the bone marrow microenvironment in multiple ways, repressing the "stemness" of cells which results in a decrease in bone formation. The number of precursor cells of the hematopoietic and osteogenic cells as well as their proliferative ability decrease with aging [10,11]. Although there are some contradictory findings in the literature related to proliferative and osteogenic potential, some of them rely on studies performed with total bone marrow population [17,26]. Other experiments [9-15], including ones in our laboratory [15,19], have focused on the mesenchymal stem cells and have shown a decrease in the stemness potential followed by a decline in the proliferative and osteogenic capacities. Consequently the molecular mechanism of the decrease in stemness associated with a decrease in bone formation with aging is yet unclear.

To better understand the molecular mechanisms governing the cellular changes we profiled the response of *ex vivo *cultured stromal cells challenged by Dex and compared cells derived from young and old rats. The gene expression profile revealed overlap of Dex action between the MSC derived from the two age groups. The functional analysis of the differentially expressed genes highlighted a general shift in the metabolism of the MSCs which included repression of proliferation and was complemented by induction of differentiation. Specifically, we monitored genes related to induction of myogenic and osteogenic differentiation coupled with a decrease in adipogenesis.

The results presented show a decrease in proliferation coupled with an increase in muscle related genes, observed in both age groups (Cluster C). The expression of muscle related genes indicates that the potential of mesenchymal stem cells to differentiate to myotubes is enhanced by Dex, which was reported earlier [27]. A reciprocal relationship of proliferation and differentiation is known – acquisition of differentiated phenotype is accompanied by a decrease in cells' proliferation potential, a relationship which is well documented for osteoblasts [28] and for myoblasts [29]. Dysfunction of tumor suppressor proteins results in attenuation of osteogenesis and myogenesis, *in vivo *[30,31] and *in vitro *[32]. It was shown that myoblasts lacking *Rb *failed to differentiate into myotubes even when transfected with *MyoD*, an essential transcription factor for myogenesis [33]. In the MSCs analyzed in the present study, Dex decreased proliferation and triggered myogenesis. In addition, the genes induced are known to play a role in osteogenesis, which was also induced. Few examples are shown by the expression of *PDGFa *[34], *TGFb3 *and *p53 *[32], genes known to play a role in osteogenesis [35] in addition to myogenic differentiation.

Aging is accompanied by a reduced stemness potential of MSCs resulting in a decrease of osteogenesis and replacement of bone marrow with fat cells. This phenomena is associated with osteoporosis [36] and overall reduction of osteogenic stem cells with aging [12]. In agreement with this, in our study the cells derived from young rats, but not the ones derived from old rats, responded to Dex with an increase in genes involved in bone remodeling, angiogenesis and a decrease in genes related to adipocyte differentiation.

Genes increased in cells derived from young rats (Cluster A) were related to bone remodeling and included *PTHr1*, *BSP*, *Madh5*, *Mepe*, *byglican *and *BMP4 *which are expressed in osteoblasts during differentiation and bone regeneration [35,37,38], indicating an anabolic effect of Dex on the cells derived from young rats. The anabolic effect of Dex is known, as demonstrated by increases in the expression of bone related genes including *BSP *in rat osteoblastic cells [39,40]. Such an effect was not observed on cells derived from old rats, which might be related to the decrease in bone formation potential recognized with aging. Neovascularization is linked to osteoblast maturation and bone deposition [41]. The angiogenesis functional group included *VEGF*, *Id1*, *Madh5*, *beta-catenin *and other genes. These genes are recognized in the literature for their role in osteoblastic differentiation in addition to their role in angiogenesis [42-45]. The expressed genes' function in angiogenesis implies that MSCs induce or attract formation of blood vessels. In addition, examining the human and mouse orthologs revealed ECM structural constituents including proteins that build up the basement membrane, like *collagens IV, V, VI *and *laminin*. Expression of these genes can indicate a potential for an endothelial differentiation.

Genes repressed following Dex treatment in cells derived from young rats (Cluster D) are related to adipocyte differentiation, regulation and function. This function included the two transcription factors *PPARγ *and *CEBPα*, which are essential for adipocyte differentiation. *PPARγ *maintains adipogenesis and is necessary for early differentiation and survival of mature adipocytes [46]. *CEBPα *is activated later than *PPARγ *during adipogenesis [47] and lack of this TF results in non-functional adipocytes [48]. These factors are sufficient for adipogenic differentiation in non-adipogenic mesenchymal cells [49] including myoblasts [50]. Adipogenesis and osteogenesis represent two divergent pathways of differentiation from the mesenchymal stem cells. Genes up-regulated in osteogenesis, such as *Msx2*, repress adipogenesis by inhibition of *PPARγ *and *CEBPα *[49]. Alternatively, PPARγ inhibits osteogenesis suppressing the osteoblastic phenotype [51]. We have demonstrated an interchanging relationship between osteogenesis and adipogenesis affected by Dex. Dex increased *beta-catenin *in the cells derived from young rats that is concurrent with an increase in osteoblastic potential of these cells. *Beta-catenin *represses adipogenesis [52] and induces osteogenesis [45]. Thus, the Dex effect of suppressing the adipogenesis (Table 2, cluster D) and increasing the osteogenesis (Table 1, cluster A) suggests that *beta-catenin *plays a role in the Dex dependent modulation of the two lineages.

The genes repressed in both ages (Cluster F, Table 2) include proliferation related genes, which complement the genes induced in cells from both age groups. We also observed a decrease in adipogenesis related genes, and in catabolic peptidases, indicating an anabolic effect of Dex. In addition, Dex decreased the expression of genes related to defense and immune response (Table 2). Dex is known to decrease the expression of immune genes, mostly tested on lymphocytes [21]. Mesenchymal cells have been shown to express immune cell markers, known to produce supportive stroma that regulates the HIM in the differentiation of the immune cells [53,54]. Thus, the decrease in immune-related genes may be related to the stromal function. This is strengthened by the fact that repressed genes also included sub-sets of membranous proteins and genes related to antigen processing and presentation (human and mouse orthologs). Hormonal modulation affects cells signaling. We identified repression of RAS pathway, which included the genes *Nras*, *Kras2*, *Grb2 *and *Aps *and also play a role in control of cell proliferation and can also repress differentiation in the myogenic [55] and osteogenic [56] pathways. *Grb2 *adaptor protein mediates the proliferative anti-myogenic differentiation action of *Met *(HGF Recptor) [57], *FGFR *[58] and is involved in signaling of Dystrophin complex [59]. The decrease in Ras signaling genes is complimentary to the proliferation and differentiation genes induced by Dex (Table 1, Cluster C) and indicates a decrease in cell proliferation, which is coupled with myoblastic or osteoblastic differentiation.

Suppression of adipogenic differentiation was noted by decrease of genes related to lipid binding, lipid transport and diacylglycerol binding including representative genes, such as *FABP-4 *(*aP-2*) [60,61] and *lipoprotein lipase *(*Lpl*) [62]. *Adipsin *repression marked the catabolism function of adipocytes [63]. Protein Kinase C isoforms are associated with various differentiation pathways. PKC repression increases myogenesis [64] and as a mediator of PTH receptor actions this enzyme is known to increase osteogenesis and repress adipogenesis in mesenchymal cell lines [65]. PKC is also associated with inducing osteoblast production of IL-6 [66] which promotes bone resorption. Thus, the decrease in PKC induces mesenchymal differentiation to the myogenic pathway.

Catabolic peptidases and lysosomal proteins decreased following Dex treatment. *Matrix metalloproteinases 7,9,12 & 13*, genes which play a role in collagen catabolism and bone remodeling [67] were included in this group. Additional genes were lysosomal proteases important in lympho-myeloid cells and cathepsins. *Cathepsin K *is the main protease in osteoclastic function that plays a role in bone resorption [68]. The decrease in expression of these genes indicates a lower rate of bone resorption, which consequently results in an increase in gene related to bone formation mediating the effects of Dex.

## Conclusion

Aging affects the bone marrow microenvironment in multiple ways, including a decrease in the differentiation potential of cells associated with decrease in bone formation. To better understand molecular mechanisms governing the cellular changes we profiled the response of *ex vivo *cultured stromal cells to Dex and compared cells derived from young and old rats. The gene expression profiling highlights age-independent modes of Dex action which overlapped between the two age groups. While this study demonstrates the effect of Dex on in *ex vivo *cultured cells, it would be interesting to expand the scope to include the *in vivo *effects of Dexamethasone. In general, pattern of expression included repression of proliferation and induction of differentiation. The RNA analyzed was extracted from pooled samples from multiple animals, a method which has been proven to reduce variability, recommended when using a small number of microarrays [69]. It is possible that additional replicates would further refine these gene lists and may alter some of our conclusions. Continuing research and verification of this interesting subject are required in the future. Specifically, we catalogued genes related to induction of myogenic differentiation coupled with a decrease in adipogenesis. We demonstrated Dex-related decrease in immune response genes; Dex decreased genes that regulate bone resorption and induced osteoblastic differentiation. The high throughput analysis enlightens the effect of Dex and the relationship between osteogenesis and adipogenesis in the bone marrow due to aging. We have also demonstrated the plasticity of cells and the reciprocal relationship of the osteogenic, myogenic and adipogenic lineages in the bone marrow.

## Methods

### *Ex vivo *cultures of mesenchymal cells

Mesenchymal stromal cells (MSCs) were cultured from female Wistar rats. Cells were cultured in Dulbecco's modified essential Medium (DMEM) with 10% heat-inactivated fetal calf serum (FCS) (Gibco, USA) [4]. The bone marrow cells were harvested from young rats (3 months old) and old rats (15 months old). Cells were collected as previously described [15]. In brief, the bone marrow cells were flushed from the long bone collected and prepared for single cells suspension. Cells pooled from six rats in each group were cultured in 75 cm^2 ^flasks (Falcon, USA) containing 20 ml of growth medium, Dulbecco's Modified Essential Medium (DMEM) containing 10% heat-inactivated fetal calf serum (FCS). Under these conditions, the hematopoietic cells died and the cultures finally remained only with cells forming the adherent stromal fibroblast-like layer. Cells were plated and after one week of culturing were trypsinized, single cell suspensions were re-cultured for 7 days and were grown in absence or presence of 10^-8 ^M Dexamethasone (Dex). On day 14 cells were harvested for RNA extraction and created four samples – young untreated (1 – YU), old untreated (2 – OU), young treated (3 – YT) and old treated cells (4 – OT).

### RNA analysis and microarrays

Total RNA were extracted from the design four experimental groups. Each group is composed from multiple animals, a method which has been proven to reduce variability, and is recommended when using a small number of microarrays [69]. RNA was extracted using the TRIzol and cRNA prepared according to Affymetrix protocols. Gene expression was measured by hybridization to Affymetrix RAE230A Gene Chip DNA microarrays (Affymetrix, Santa Clara, CA, USA), containing 15,766 probe sets (PS) (excluding controls), comprising 14,280 Unigene clusters (11,497 Gene IDs, 4,699 full-length transcripts). The data is available as accession GSE3339 of the Gene Expression Omnibus (GEO).

### Gene expression analysis

Total RNA extracted from cultured cells following ex vivo expansion was reverse transcribed using avian myeloblastosis virus reverse transcriptase (AMV-RT) and oligo-dT to generate cDNA that served as a template for the polymerase chain reaction (PCR) (Takara Shuzo Co. Ltd., Japan) with gene specific primers (Table 4). The integrity of the RNA, the efficiency of the RT reaction and the quality of cDNA subjected to the RT-PCR was controlled by amplification of Gluteraldehyde-3-Phosphate Dehydrogenase (G3PDH) (Clontech, Palo Alto, CA). The reaction products were separated by electrophoresis in 1% agarose gels (SeaKem GTG, FMC, USA) in Tris Borate EDTA (TBE) buffer. The amplified DNA fragments were stained by ethidium bromide, and their optical density was measured using Bio Imaging System, BIS 202D and analyzed using "TINA" software. PCR amplification was performed at least twice and subjected to semi-quantitative analyses by comparison of OD of gene-specific PCR products normalized to the OD of co-amplified G3PDH-PCR product in four groups YO, OU, YT and OT.

### The computerized data analysis

We used the MAS 5.0 algorithm to provide a baseline expression level and detection for each PS. PS were filtered according to the following criteria: (a) at least one sample was detected as Present (P) when calculating MAS 5.0 detection call; and (b) at least one sample had an expression level higher than 20. The expression levels of 10290 retained PS normalized using the Quantile Normalization method, provided by the software package 'affy' (version 1.5.8) [70], available as part of the Bioconductor project. For every PS, the ratio between expression level of the treated and untreated samples was calculated. PS were analyzed when the expression ratio at least 2 fold, in either age group. Gene expression of cells cultured from young rats (young treated versus young untreated) was compared to cells from old rats (old treated versus old untreated). After filtering the PS were standardized by transforming each expression pattern to have a mean of 0 and a variance of 1.

### Clustering analysis

We used a rule-based clustering method on the PS that were differentially expressed when the fold change was at least two and were analyzed for either increasing or decreasing expression between Dex-treated and untreated cells. For every PS, the ratio between expression level of the treated and untreated samples was calculated. Every PS was graded as increased, decreased or unchanged. The young grade (YT/YU) was compared to the old grade (OT/OU), and this determined the cluster. This method resulted in 4060 PS, clustered in 8 distinct clusters. This clustering method separated increased and decreased PS due to Dex, and highlighted difference between old and young rats. Partial data is discussed in this paper and complete lists of functions and genes are available upon request.

### Gene Ontology analysis

The clusters were analyzed for Gene Ontology (GO) annotations appearing in a statistically significant manner in the cluster, when compared to a background. The background was comprised of all expressed genes – a list of all genes expressed in at least one of the four samples. For each GO term the number of genes with this term in a cluster was compared to the number of genes with this term in the background. Additionally the ratio between number of genes in the cluster and number of gene in the background was calculated. The two ratios served to calculate a p-value, and terms with a p-value lower than 0.01 were listed. The analysis was done using GO Tree Machine (GOTM) [71], the 08/09/04 version. For verification, the same functional analysis was done on Human and Mouse orthologs (provided by Affymetrix 29/07/04), using the EXPANDER software suite [72].

## Authors' contributions

UDA carried out the computational analysis, IS carried out the molecular studies. DB conceived of the study, designed and coordinated it. The GeneChip hybridization was performed at the Functional Genomics unit, The Chaim Sheba Medical Center and Sackler School of Medicine headed by GR. All authors helped to draft the manuscript, read and approved the final manuscript.

## Figures and Tables

**Table 1:** Genes induced by Dexamethasone in MSCs

Function	p-value	Up to fold change	Number of Genes
Cluster A – Dex induced genes in MSCs derived from young rats			
Angiogenesis	0.00125	5	5
Bone remodeling	0.00448	2	4
Cluster C – Dex induced genes in MSCs derived from young and old rats			
Cell growth	2.36E-06	5	11
Regulation of cell cycle	0.00418	4	13
Muscle development	0.00555	4	7
Muscle contraction	0.00157	4	4
Cytoskeleton	0.00868	2	19
Actin cytoskeleton	0.00803	4	7
Structural constituent of cytoskeleton	0.00533	4	4

**Table 2:** Genes repressed by Dexamethasone in MSCs

Function	p-value	Up to fold change	Number of Genes
Cluster D – Dex repressed genes in MSCs derived from young rats			
Adipocyte differentiation	0.00563	2	2
Cluster F – Dex repressed genes in MSCs derived from young & old rats			
RAS protein signal transduction	0.00016	4	4
Defense response	0.00060	5	20
Catabolism	0.00027	4	31
Peptidase activity	0.00282	4	21
Lipid binding	0.00024	5	12
Diacylglycerol binding	0.00474	2	5
Lipid transport	0.00960	3	5
Hydrolase activity	0.00019	4	55
Lysosome vacuole	1.4E-07	5	13

**Table 3:** Gene array analysis for adipogenesis, osteogenesis and myogenesis pathways

GeneID	Name	Probe_ID	Fold change	
			
			YT/YU	OT/OU
**Cluster A**				
*Angiogenesis*				
114111 ^a^	vascular endothelial growth factor	1368463_at	3.452121	1.502053
24617 ^a^	Serine/cysteine proteinase inhibitor1	1368519_at	5.822078	0.845776
25261 ^a^	Inhibitor of DNA binding 1, helix-loop-helix protein	1387028_a_at	3.49452	0.986531
29452 ^a^	Endothelial PAS domain protein 1	1369703_at	5.615	1
64032 ^a^	connective tissue growth factor	1367631_at	2.58613	1.079194
84353 ^b^	beta-catenin	1369733_at	2.459261	1.906824
*Bone Remodeling*				
24477	integrin binding sialoprotein	1368416_at	7.7875	1
56813	parathyroid hormone receptor 1	1370259_a_at	2.66125	1.123853
59328	MAD homolog 5 (Drosophila)*	1369276_at	2.14625	1.970041
79110	matrix extracellular phosphoglycoprotein	1387330_at	3.37375	0.997506
**Cluster C**				
*Cytoskeleton & Muscle Development*				
24582 ^ac^	myosin heavy chain 11	1370896_a_at	5.571237	4.653653
24851 ^ac^	tropomyosin 1, alpha	1370288_a_at	4.89204	5.13257
25365	Actin, gamma 2	1386869_at	4.126485	5.083472
29437 ^ac^	Actin alpha 1	1369928_at	17.85533	9.397908
64362 ^ac^	Desmin	1367600_at	2.398671	2.356318
114489 ^d^	dystroglycan 1	1389105_at	5.441386	7.678867
303468 ^bd^	Sarcoglycan	1372240_at	2.9525	2.708798
25485	myosin IC	1369779_at	5.11	2.61875
81531	profilin II	1367970_at	3.62625	4.4225
24170	Adducin 1, alpha	1388487_at	2.475238	2.510223
*Cell growth & Cell Cycle*				
24842	tumor protein p53	1367830_a_at	2.264291	2.543204
64457 ^d^	N-myc downstream regulated 4	1370229_at	4.279081	3.103067
29576 ^d^	WNT1 inducible signaling pathway protein 2	1369484_at	7.956218	2.918245
24183	adenylate kinase 1	1370011_at	4.118338	4.754549
25163	cyclin dependent kinase inhibitor 2A	1369194_a_at	14.86273	8.253055
50594	neuroblastoma, suppression of tumorigenicity 1	1387004_at	3.827506	2.863581
58919	cyclin D1	1371150_at	3.147613	2.447929
64363	v-raf murine sarcoma 3611 viral oncogene homolog 1	1388305_at	2.02	2.018793
83570	c-fos induced growth factor (vascular endothelial growth factor D)	1387709_at	11.6725	3.355
94201	cyclin-dependent kinase 4	1372739_at	2.388806	2.154783
**Cluster D**				
*Adipocyte Differentiation*				
24252	CCAAT/enhancer binding protein, alpha	1369658_at	0.460345	0.627943
25664	peroxisome proliferator activated receptor, gamma	1369179_a_at	0.291579	0.79108
**Cluster F**				
*Lipid Binding & Transport*				
140868	fatty acid binding protein 5	1370281_at	0.144064	0.4143
170538	protein kinase C, delta	1387114_at	0.365549	0.470085
25023	protein kinase C, beta 1	1370585_a_at	0.028032	0.061769
25056	retinol binding protein 1	1367939_at	0.468695	0.471976
25728	apolipoprotein E	1370862_at	0.308694	0.087332
25292	apolipoprotein C-I	1368587_at	0.175235	0.329354
29184	cd36 antigen	1386901_at	0.055742	0.035525
29510	phosphatidylcholine transfer protein	1387058_at	0.346607	0.226442
79124	ZAP 36/annexin IV	1389305_at	0.488443	0.480585
79451	adipocyte protein aP2	1368271_a_at	0.039507	0.01239
*Catabolism & Lysosomal Enzymes*				
117033 ^ac^	matrix metalloproteinase 12	1368530_at	0.02401	0.214259
171052 ^ac^	matrix metalloproteinase 13	1388204_at	0.155018	0.195806
25335 ^ac^	matrix metalloproteinase 7	1368766_at	0.150338	0.189753
24331 ^ac^	elastase 1	1387819_at	0.108743	0.2376
25166 ^ac^	caspase 1	1369186_at	0.255206	0.175345
171329 ^ac^	ubiquitin specific protease 15	1370460_at	0.295028	0.444408
24539 ^ac^	lipoprotein lipase	1386965_at	0.016611	0.039975
54249 ^bd^	Adipsin	1388602_at	0.18685	0.047754
313387 ^bd^	ubiquitin specific protease 1	1376687_at	0.417153	0.373377
24944 ^ac^	lysosomal membrane glycoprotein 2	1370010_at	0.430907	0.347294
*Ras protein signal transduction*				
114203	adaptor protein with pleckstrin homology and src homology 2 domains	1368605_at	0.2262	0.175548
24525	Kirsten rat sarcoma viral oncogene homologue 2 (active)	1370035_at	0.312723	0.375405
24605	neuroblastoma RAS viral (v-ras) oncogene homolog	1372032_at	0.191657	0.238384
81504	growth factor receptor bound protein 2	1368386_at	0.373241	0.484997
*Defense Response*				
113959	complement component 5, receptor 1	1368742_at	0.234295	0.239768
116591	Fc receptor, IgG, low affinity III	1367850_at	0.292948	0.310885
24223 ^a^	beta-2 microglobulin	1371440_at	0.13671	0.16094
24494 ^ac^	interleukin 1 beta	1398256_at	0.055097	0.172791
56822 ^ac^	CD86 antigen	1387627_at	0.206497	0.402268
25599 ^ac^	CD74 antigen	1367679_at	0.011698	0.040091
60350 ^ac^	CD14 antigen	1368490_at	0.469431	0.412767
24930 ^a^	CD8 antigen, alpha chain	1369877_at	0.36815	0.4273
24931 ^a^	CD8 antigen, beta chain	1387739_at	0.203248	0.376336
24932 ^ac^	CD4 antigen	1369483_at	0.204226	0.301498

The fold change are of treated versus untreated cells from young (3 month) or old (15 month) rats. In genes that have multiple probe sets, a representative probe set was displayed.

a) gene was selected with the relevant function in both rat genes and mouse orthologs

b) gene was selected with the relevant function in mouse orthologs,

c) gene was selected with the relevant function in both rat genes and human orthologs

d) gene was selected with the relevant function in human orthologs,

*) MAD homolog 5 appears also under the function of angiogenesis

**Table 4:** Primers used for RT-PCR amplification

Gene	Primers	Size of PCR Product
ADIPSIN	F: GAGGCGGCTGTATGTGTTG R: AGGCATTGTGGGAGAGCTTA	337 bp
LPL	F: CCCTAAGGACCCCTGAAGAC R: AGCTGGATCCAAGCCAGTAA	401 bp
BIGLYCCAN	F: TGATGAGGAGGCTTCAGGTT R: ACTTTGCGGATACGGTTGTC	413 bp
BMP4	F: ATGAGGGATCTTTACCGGCT R: TTTATACGGTGGAAGCCCTG	293 bp
BSP	F: AGGGGAATGAAGACCAGGAG R: TTCGTCCTCATAAGCTCGGTA	607 bp
GR	F: ACCACAGACCAAAGCACCTT R: AAGGGATGCTGTATTCATGTCA	444 bp
DESMIN	F: CCGAGCTCTACGAGGAGGA R: ACCTGCTGTTCCTGAAGCTG	321 bp
ACTIN GAMMA2	F: GGAGAAGATCTGGCACCACT R: TGATCCACATTTGCTGGAAG	826 bp
AP-2	F: TGTCTCCAGTGAAAACTTCGATGA R: ACTCTTGTGGAAGTCACGCCTTT	350 bp
G3PDH	F: ACCACAGTCCATGCCATCAC R: TCCACCACCCTGTTGCTGTA	450 bp

## References

[B1] Benayahu D (2000). The Hematopoietic Microenvironment: The Osteogenic Compartment of Bone Marrow: Cell Biology and Clinical Application. Hematol.

[B2] Friedenstein AJ, Ivanov-Smolenski AA, Chajlakjan RK, Gorskaya UF, Kuralesova AI, Latzinik NW, Gerasimow UW (1978). Origin of bone marrow stromal mechanocytes in radiochimeras and heterotopic transplants. Exp Hematol.

[B3] Owen M, Friedenstein AJ (1988). Stromal stem cells: marrow-derived osteogenic precursors. Ciba Found Symp.

[B4] Benayahu D, Kletter Y, Zipori D, Wientroub S (1989). Bone marrow-derived stromal cell line expressing osteoblastic phenotype in vitro and osteogenic capacity in vivo. J Cell Physiol.

[B5] Haynesworth SE, Goshima J, Goldberg VM, Caplan AI (1992). Characterization of cells with osteogenic potential from human marrow. Bone.

[B6] Triffitt JT, Joyner CJ, Oreffo RO, Virdi AS (1998). Osteogenesis: bone development from primitive progenitors. Biochem Soc Trans.

[B7] Aubin JE (2001). Regulation of osteoblast formation and function. Rev Endocr Metab Disord.

[B8] Kirschstein R, Skirboll LR Stem Cells: Scientific Progress and Future Research Directions. http://stemcells.nih.gov/info/scireport.

[B9] Kahn A, Gibbons R, Perkins S, Gazit D (1995). Age-related bone loss. A hypothesis and initial assessment in mice. Clin Orthop Relat Res.

[B10] Bergman RJ, Gazit D, Kahn AJ, Gruber H, McDougall S, Hahn TJ (1996). Age-related changes in osteogenic stem cells in mice. J Bone Miner Res.

[B11] Oreffo RO, Bennett A, Carr AJ, Triffitt JT (1998). Patients with primary osteoarthritis show no change with ageing in the number of osteogenic precursors. Scand J Rheumatol.

[B12] D'Ippolito G, Schiller PC, Ricordi C, Roos BA, Howard GA (1999). Age-related osteogenic potential of mesenchymal stromal stem cells from human vertebral bone marrow. J Bone Miner Res.

[B13] Katzburg S, Lieberherr M, Ornoy A, Klein BY, Hendel D, Somjen D (1999). Isolation and hormonal responsiveness of primary cultures of human bone-derived cells: gender and age differences. Bone.

[B14] Nishida S, Endo N, Yamagiwa H, Tanizawa T, Takahashi HE (1999). Number of osteoprogenitor cells in human bone marrow markedly decreases after skeletal maturation. J Bone Miner Metab.

[B15] Liu Z, Graff E, Benayahu D (2000). Effect of raloxifene-analog (LY 117018-Hcl) on the bone marrow of ovariectomized mice. J Cell Biochem.

[B16] Bellows CG, Pei W, Jia Y, Heersche JN (2003). Proliferation, differentiation and self-renewal of osteoprogenitors in vertebral cell populations from aged and young female rats. Mech Ageing Dev.

[B17] Stenderup K, Justesen J, Clausen C, Kassem M (2003). Aging is associated with decreased maximal life span and accelerated senescence of bone marrow stromal cells. Bone.

[B18] Moerman EJ, Teng K, Lipschitz DA, Lecka-Czernik B (2004). Aging activates adipogenic and suppresses osteogenic programs in mesenchymal marrow stroma/stem cells: the role of PPAR-gamma2 transcription factor and TGF-beta/BMP signaling pathways. Aging Cell.

[B19] Benayahu D, Shur I, Ben-Eliyahu S (2000). Hormonal changes affect the bone and bone marrow cells in a rat model. J Cell Biochem.

[B20] Shur I, Lokiec F, Bleiberg I, Benayahu D (2001). Differential gene expression of cultured human osteoblasts. J Cell Biochem.

[B21] Czock D, Keller F, Rasche FM, Haussler U (2005). Pharmacokinetics and pharmacodynamics of systemically administered glucocorticoids. Clin Pharmacokinet.

[B22] Cheng SL, Yang JW, Rifas L, Zhang SF, Avioli LV (1994). Differentiation of human bone marrow osteogenic stromal cells in vitro: induction of the osteoblast phenotype by dexamethasone. Endocrinology.

[B23] Fried A, Benayahu D, Wientroub S (1993). Marrow stroma-derived osteogenic clonal cell lines: putative stages in osteoblastic differentiation. J Cell Physiol.

[B24] Fried A, Benayahu D (1996). Dexamethasone regulation of marrow stromal-derived osteoblastic cells. J Cell Biochem.

[B25] Kuznetsov SA, Krebsbach PH, Satomura K, Kerr J, Riminucci M, Benayahu D, Robey PG (1997). Single-colony derived strains of human marrow stromal fibroblasts form bone after transplantation in vivo. J Bone Miner Res.

[B26] Xu CX, Hendry JH, Testa NG, Allen TD (1983). Stromal colonies from mouse marrow: characterization of cell types, optimization of plating efficiency and its effect on radiosensitivity. J Cell Sci.

[B27] Grigoriadis AE, Heersche JN, Aubin JE (1988). Differentiation of muscle, fat, cartilage, and bone from progenitor cells present in a bone-derived clonal cell population: effect of dexamethasone. J Cell Biol.

[B28] Stein GS, Lian JB, Owen TA (1990). Relationship of cell growth to the regulation of tissue-specific gene expression during osteoblast differentiation. Faseb J.

[B29] Walsh K, Perlman H (1997). Cell cycle exit upon myogenic differentiation. Curr Opin Genet Dev.

[B30] Zhang P, Wong C, Liu D, Finegold M, Harper JW, Elledge SJ (1999). p21(CIP1) and p57(KIP2) control muscle differentiation at the myogenin step. Genes Dev.

[B31] Takahashi K, Nakayama K (2000). Mice lacking a CDK inhibitor, p57Kip2, exhibit skeletal abnormalities and growth retardation. J Biochem (Tokyo).

[B32] Ghosh-Choudhury N, Harris MA, Wozney J, Mundy GR, Harris SE (1997). Clonal osteoblastic cell lines from p53 null mouse calvariae are immortalized and dependent on bone morphogenetic protein 2 for mature osteoblastic phenotype. Biochem Biophys Res Commun.

[B33] Novitch BG, Spicer DB, Kim PS, Cheung WL, Lassar AB (1999). pRb is required for MEF2-dependent gene expression as well as cell-cycle arrest during skeletal muscle differentiation. Curr Biol.

[B34] Fiedler J, Etzel N, Brenner RE (2004). To go or not to go: Migration of human mesenchymal progenitor cells stimulated by isoforms of PDGF. J Cell Biochem.

[B35] Favus MJ (2003). Primer on the metabolic bone diseases and disorders of mineral metabolism.

[B36] Nuttall ME, Gimble JM (2000). Is there a therapeutic opportunity to either prevent or treat osteopenic disorders by inhibiting marrow adipogenesis?. Bone.

[B37] Petersen DN, Tkalcevic GT, Mansolf AL, Rivera-Gonzalez R, Brown TA (2000). Identification of osteoblast/osteocyte factor 45 (OF45), a bone-specific cDNA encoding an RGD-containing protein that is highly expressed in osteoblasts and osteocytes. J Biol Chem.

[B38] Lu C, Huang S, Miclau T, Helms JA, Colnot C (2004). Mepe is expressed during skeletal development and regeneration. Histochem Cell Biol.

[B39] Porter RM, Huckle WR, Goldstein AS (2003). Effect of dexamethasone withdrawal on osteoblastic differentiation of bone marrow stromal cells. J Cell Biochem.

[B40] Atmani H, Chappard D, Basle MF (2003). Proliferation and differentiation of osteoblasts and adipocytes in rat bone marrow stromal cell cultures: effects of dexamethasone and calcitriol. J Cell Biochem.

[B41] Carano RA, Filvaroff EH (2003). Angiogenesis and bone repair. Drug Discov Today.

[B42] Dai J, Kitagawa Y, Zhang J, Yao Z, Mizokami A, Cheng S, Nor J, McCauley LK, Taichman RS, Keller ET (2004). Vascular endothelial growth factor contributes to the prostate cancer-induced osteoblast differentiation mediated by bone morphogenetic protein. Cancer Res.

[B43] Maeda Y, Tsuji K, Nifuji A, Noda M (2004). Inhibitory helix-loop-helix transcription factors Id1/Id3 promote bone formation in vivo. J Cell Biochem.

[B44] Liu B, Mao N (2004). Smad5: signaling roles in hematopoiesis and osteogenesis. Int J Biochem Cell Biol.

[B45] Hu H, Hilton MJ, Tu X, Yu K, Ornitz DM, Long F (2005). Sequential roles of Hedgehog and Wnt signaling in osteoblast development. Development.

[B46] Knouff C, Auwerx J (2004). Peroxisome Proliferator-Activated Receptor-{gamma} Calls for Activation in Moderation: Lessons from Genetics and Pharmacology. Endocr Rev.

[B47] Rosen ED, Walkey CJ, Puigserver P, Spiegelman BM (2000). Transcriptional regulation of adipogenesis. Genes Dev.

[B48] Darlington GJ, Ross SE, MacDougald OA (1998). The role of C/EBP genes in adipocyte differentiation. J Biol Chem.

[B49] Ichida F, Nishimura R, Hata K, Matsubara T, Ikeda F, Hisada K, Yatani H, Cao X, Komori T, Yamaguchi A, Yoneda T (2004). Reciprocal roles of MSX2 in regulation of osteoblast and adipocyte differentiation. J Biol Chem.

[B50] Hu E, Tontonoz P, Spiegelman BM (1995). Transdifferentiation of Myoblasts by the Adipogenic Transcription Factors PPAR{gamma} and C/EBP{alpha}. PNAS.

[B51] Akune T, Ohba S, Kamekura S, Yamaguchi M, Chung UI, Kubota N, Terauchi Y, Harada Y, Azuma Y, Nakamura K, Kadowaki T, Kawaguchi H (2004). PPARgamma insufficiency enhances osteogenesis through osteoblast formation from bone marrow progenitors. J Clin Invest.

[B52] Moldes M, Zuo Y, Morrison RF, Silva D, Park BH, Liu J, Farmer SR (2003). Peroxisome-proliferator-activated receptor gamma suppresses Wnt/beta-catenin signalling during adipogenesis. Biochem J.

[B53] Barda-Saad M, Shav-Tal Y, Rozenszajn AL, Cohen M, Zauberman A, Karmazyn A, Parameswaran R, Schori H, Ashush H, Ben-Nun A, Zipori D (2002). The mesenchyme expresses T cell receptor mRNAs: relevance to cell growth control. Oncogene.

[B54] Barda-Saad M, Zhang AS, Zipori D, Rozenszajn LA (1997). Adhesion of thymocytes to bone marrow stromal cells: regulation by bFGF and IFN-gamma. Stem Cells.

[B55] Olson EN, Spizz G, Tainsky MA (1987). The oncogenic forms of N-ras or H-ras prevent skeletal myoblast differentiation. Mol Cell Biol.

[B56] Tanaka Y, Nakayamada S, Fujimoto H, Okada Y, Umehara H, Kataoka T, Minami Y (2002). H-Ras/Mitogen-activated Protein Kinase Pathway Inhibits Integrin-mediated Adhesion and Induces Apoptosis in Osteoblasts. J Biol Chem.

[B57] Leshem Y, Gitelman I, Ponzetto C, Halevy O (2002). Preferential binding of Grb2 or phosphatidylinositol 3-kinase to the met receptor has opposite effects on HGF-induced myoblast proliferation. Exp Cell Res.

[B58] Kontaridis MI, Liu X, Zhang L, Bennett AM (2002). Role of SHP-2 in fibroblast growth factor receptor-mediated suppression of myogenesis in C2C12 myoblasts. Mol Cell Biol.

[B59] Oak SA, Zhou YW, Jarrett HW (2003). Skeletal muscle signaling pathway through the dystrophin glycoprotein complex and Rac1. J Biol Chem.

[B60] Tan NS, Shaw NS, Vinckenbosch N, Liu P, Yasmin R, Desvergne B, Wahli W, Noy N (2002). Selective Cooperation between Fatty Acid Binding Proteins and Peroxisome Proliferator-Activated Receptors in Regulating Transcription. Mol Cell Biol.

[B61] Glatz JFC, Borchers T, Spener F, van der Vusse GJ (1995). Fatty acids in cell signalling: Modulation by lipid binding proteins. Prostaglandins, Leukotrienes and Essential Fatty Acids.

[B62] Mead JR, Irvine SA, Ramji DP (2002). Lipoprotein lipase: structure, function, regulation, and role in disease. Journal of Molecular Medicine.

[B63] Cook KS, Groves DL, Min HY, Spiegelman BM (1985). A Developmentally Regulated mRNA from 3T3 Adipocytes Encodes a Novel Serine Protease Homologue. PNAS.

[B64] Goel HL, Dey CS (2002). PKC-regulated myogenesis is associated with increased tyrosine phosphorylation of FAK, Cas, and paxillin, formation of Cas-CRK complex, and JNK activation. Differentiation.

[B65] Chan GK, Miao D, Deckelbaum R, Bolivar I, Karaplis A, Goltzman D (2003). Parathyroid Hormone-Related Peptide Interacts with Bone Morphogenetic Protein 2 to Increase Osteoblastogenesis and Decrease Adipogenesis in Pluripotent C3H10T1/2 Mesenchymal Cells. Endocrinology.

[B66] Nagy Z, Radeff J, Stern PH (2001). Stimulation of interleukin-6 promoter by parathyroid hormone, tumor necrosis factor alpha, and interleukin-1beta in UMR-106 osteoblastic cells is inhibited by protein kinase C antagonists. J Bone Miner Res.

[B67] Geoffroy V, Marty-Morieux C, Le Goupil N, Clement-Lacroix P, Terraz C, Frain M, Roux S, Rossert J, de Vernejoul MC (2004). In vivo inhibition of osteoblastic metalloproteinases leads to increased trabecular bone mass. J Bone Miner Res.

[B68] Lindeman JH, Hanemaaijer R, Mulder A, Dijkstra PD, Szuhai K, Bromme D, Verheijen JH, Hogendoorn PC (2004). Cathepsin K is the principal protease in giant cell tumor of bone. Am J Pathol.

[B69] Kendziorski C, Irizarry RA, Chen KS, Haag JD, Gould MN (2005). On the utility of pooling biological samples in microarray experiments. Proc Natl Acad Sci U S A.

[B70] Gautier L, Cope L, Bolstad BM, Irizarry RA (2004). affy--analysis of Affymetrix GeneChip data at the probe level. Bioinformatics.

[B71] Gentleman RC, Carey VJ, Bates DM, Bolstad B, Dettling M, Dudoit S, Ellis B, Gautier L, Ge Y, Gentry J, Hornik K, Hothorn T, Huber W, Iacus S, Irizarry R, Leisch F, Li C, Maechler M, Rossini AJ, Sawitzki G, Smith C, Smyth G, Tierney L, Yang JY, Zhang J (2004). Bioconductor: open software development for computational biology and bioinformatics. Genome Biol.

[B72] Zhang B, Schmoyer D, Kirov S, Snoddy J (2004). GOTree Machine (GOTM): a web-based platform for interpreting sets of interesting genes using Gene Ontology hierarchies. BMC Bioinformatics.

[B73] Sharan R, Maron-Katz A, Shamir R (2003). CLICK and EXPANDER: a system for clustering and visualizing gene expression data. Bioinformatics.

